# Unraveling the Biological Properties of Whey Peptides and Their Role as Emerging Therapeutics in Immune Tolerance

**DOI:** 10.3390/nu17060938

**Published:** 2025-03-07

**Authors:** Laura Quintieri, Anna Luparelli, Leonardo Caputo, William Schirinzi, Federica De Bellis, Leonardo Smiriglia, Linda Monaci

**Affiliations:** 1 Institute of Sciences of Food Production, National Research Council (ISPA-CNR), Via G. Amendola, 122/O, 70126 Bari, Italy; laura.quintieri@ispa.cnr.it (L.Q.); anna.luparelli@ispa.cnr.it (A.L.); leonardo.caputo@ispa.cnr.it (L.C.); williamschirinzi99@gmail.com (W.S.); federicadebellis.0060@gmail.com (F.D.B.); 2 Parafarmacia Smiriglia Leonardo, Via San Giorgio 19/B, 70019 Bari, Italy; smiriglialeonardo@libero.it

**Keywords:** whey proteins, bioactive peptides, health-promoting functions, non-communicable diseases, food industries, pharmaceuticals, immunotolerance, milk allergens, tolerogenic effect

## Abstract

Whey is a natural by-product of the cheese-making process and represents a valuable source of nutrients, including vitamins, all essential amino acids and proteins with high quality and digestibility characteristics. Thanks to its different techno-functional characteristics, such as solubility, emulsification, gelling and foaming, it has been widely exploited in food manufacturing. Also, advances in processing technologies have enabled the industrial production of a variety of whey-based products exerting biological activities. The beneficial properties of whey proteins (WPs) include their documented effects on cardiovascular, digestive, endocrine, immune and nervous systems, and their putative role in the prevention and treatment of non-communicable diseases (NCDs). In this regard, research on their application for health enhancement, based on the optimization of product formulation and the development of pharmaceuticals, is highly relevant. Beyond the health and nutritionally relevant effects as in in vivo animal studies, the allergenicity of WPs and WP hydrolysates is also herein tackled and discussed, as well as their potential role as therapeutics for immune tolerance and so-called tolerogenic effects. Grounded on the WPs’ health-promoting functions, this paper presents the latest research showing the potential of whey-derived peptides as an alternative strategy in NCD treatment. This work also reports a careful analysis of their current use, also revealing which obstacles limit their full exploitation, thus highlighting the future challenges in the field. Concluding, safety considerations, encompassing WP allergenicity, are also discussed, providing some insights on the role of WPs and peptides in milk allergen immunotolerance.

## 1. Introduction

**Figure 1:**
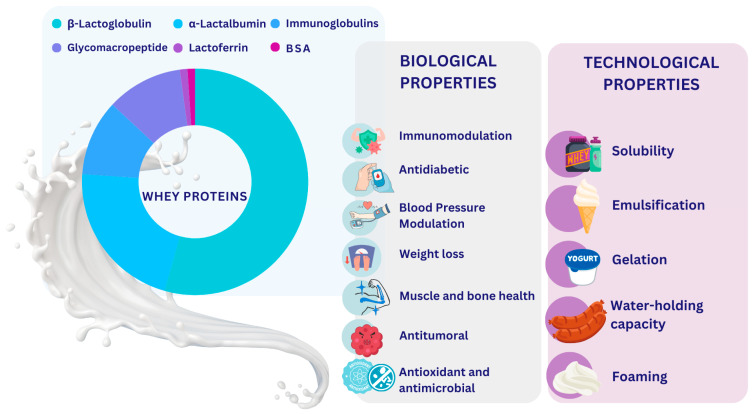
Whey proteins and main biological and technological properties.

Whey proteins (WPs) are predominantly composed of β-lactoglobulin (β-LG) and α-lactalbumin (α-LA), along with minor fractions, including bovine serum albumin (BSA), immunoglobulins (IGs) and lactoferrin (LF), and have a high biological value, representing a rich source of essential and branched-chain amino acids [1,2,3]. The nutritional and physiological benefits of WP-based foods are well known, as previously documented [4,5,6]. In addition to their remarkable nutritional properties, WPs display significant techno-functional characteristics, including solubility, emulsification, gelling and foaming properties, as depicted in Figure 1, which are of considerable value in food formulation [2,3]. WP processing by conventional methods (hydrolysis, fermentation) and green technologies (ultrasound, microwave, hydrostatic pressure, pulsed electric field and subcritical water) release peptides with healthy properties [7]; recently, in silico approaches have been used to discover and investigate the biological activity of bioactive peptides (BAPs), increasing their yield and activity, improving their stability and reducing time for their production, identification and separation [8].

The discovery of natural BAPs to be exploited in drug development, as well as their production at a larger scale, is a current challenge [9]; toward this aim, several food matrices have been investigated [10,11] and biotechnological advances have been pursued (e.g., expression systems; [12,13]).

BAPs from whey proteins are endowed with several biological activities, such as antimicrobial, antihypertensive, antithrombotic, anticancer, antioxidant, opioid, immunomodulatory, mineral-binding and regulation of gut microbiota [4,14,15,16,17,18,19,20,21,22]; recent evidence also reports effects on the central nervous system, such as anti-inflammatory and antioxidant activity on glia and astrocytes [23,24]. They can enter the body in an intact form and exhibit their activities directly in the gastrointestinal tract, as well as being a precursor of shorter sequences with similar or higher activity. Biological effects of foliar spray treatments with WP hydrolysates were also assayed in agriculture to promote the growth and increase the yield of pea plants cultivated in a clay loam soil [25].

The mechanisms of action of BAPs include regulation of gene expression and protein synthesis, interaction with nucleosome and histone proteins, single-stranded and double-stranded DNA and free radicals, as well as metal chelation [26]; BAPs can also regulate DNA methylation status, an epigenetic mechanism of gene activation or repression involved in some diseases and aging [27]. BAPs from bovine whey proteins have been extensively reported in the literature, while research on novel bioactive sequences from alternative sources, such as sheep and goat, is gaining increasing attention [16,28,29,30].

Non-communicable diseases (NCDs), also referred to as chronic diseases, mainly encompass cardiovascular conditions (such as heart attacks and stroke), cancers, chronic respiratory diseases (including chronic obstructive pulmonary disease and asthma) and diabetes; NCDs accounted for the greatest proportion of total deaths (75%) in 2021, equivalent to 43 million people around the world [31].

Several studies have shown that BAPs derived from the proteins of various food matrices (such as bean, corn, fish) have the potential to target therapeutic pathways associated with NCDs, offering novel applications in nutraceutical and pharmaceutical industries [32,33,34].

The literature reports evidence on the application of WP-derived peptides in the prevention and treatment of NCDs; however, their promising potential appears to still not be fully exploited.

In order to highlight recent advances in BAPs from whey proteins, this review will discuss in depth the novel insights into their biological and multi-target activities with special regard to the prevention and treatment of NCDs. BAPs released from non-bovine WPs will be preferred. Applications as value-added food ingredients, supplements or drugs in the food and pharmaceutical sectors will also be summarized, as well as safety considerations about their allergenicity risk.

## 2. Prevention and Treatment of Non-Communicable Diseases (NCDs)

BAPs from WPs are considered as a promising strategy for the management of most NCDs [35,36,37].

The prevalence of NCD risk factors tends to be higher among elders and in people with a sedentary lifestyle [38]. Nutrition during early life is also linked to the risk of chronic disease development [39]. In the following paragraph, the recent advances in biological properties of BAPs will be discussed in detail. Taking into account the significant and extensive literature at our disposal on whey peptides, in this review, we focused on a selection of papers more recently published and spanning the last two decades that also focus on in vivo studies (also summarized in Table 1) and on selected peptides obtained from proteins of minor mammal species.

### 2.1. Cardiovascular and Metabolic Diseases

It has been reported that BAPs can exert cardioprotective effects in cardiovascular diseases by lowering high blood pressure or hypertension thanks to the inhibition of the angiotensin-converting enzyme (ACE), involved in the renin–angiotensin system [16,17,40,41]. A cardioprotective effect has been successfully proved in vitro [17,42,43] and in vivo in hypertensive rats [40,44,45,46]. By contrast, results from clinical trials showed a reduction in their efficacy upon oral intake putatively due to gastrointestinal hydrolysis, strictly depending on their hydrophobicity, acid/base nature, amino acid composition and peptide sequence [40,47,48]. Current challenges are focused on the protection of the activity of orally administrated BAPs by the development of encapsulation methods [40] and hydrolysis protocols to increase activity and yield [49,50], for example, for the discovery of novel sequences with higher activity from WPs of minor species [16,51].

Peptides can mediate their antihypertensive properties thanks to additional mechanisms, such as inflammation modulation (TNF-α and other cytokine signaling), oxidative stress (Keap-1/Nrf2/ARE/HO-1 and related signaling pathways), PPAR-γ/caspase3/MAPK signaling pathways and inhibition of lipid accumulation [52].

The amelioration of inflammation was obtained by the inhibition of IL-1β, COX-2, TNF-α, IL-6 and the signal molecular of NK-kB pathway [53].

The nuclear factor-erythroid 2-related factor 2 (Nrf2) is the master regulator of redox hemostasis, and can resist oxidative damage by activating downstream antioxidant proteins such as heme oxygenase 1 (HO-1) and promoting antioxidant enzymes such as catalase (CAT), superoxide dismutase (SOD) and glutathione peroxidase (GSH-Px). Recently, three antioxidant peptides, PKYPVEPF, LEASPEVI and YPFPGPIHNS, obtained by fermenting WPs by *Lactobacillus rhamnosus* B2-1, demonstrated cytoprotective effects on HepG2 cells under oxidative stress induced by H_2_O_2_, increased catalase and superoxide dismutase activities and reduced reactive oxygen species levels in damaged cells. In addition, the molecular docking studies highlighted that these antioxidant peptides also prevented the interaction between Keap1 and Nrf2 [54].

Examples of WP hydrolysates endowed with antioxidant activity were reported in several studies [36,55,56,57,58]. In addition antioxidant peptides also exhibited multi-target activities [17].

A novel DPP-IV inhibitory peptide, LDQWLCEKL (IC50: 131 μM), was isolated from bovine whey α-LA (f115–123) upon trypsin hydrolysis. Similar activity was registered for ELKDLKGY and ILDKVGINY obtained by the digestion of bovine α-LA with alcalase; these results suggested that these peptides may represent a promising strategy for preventive or adjunctive therapy in the management of type 2 diabetes [36]; also, pepsi-digested β-LG contained the DPP-IV-inhibiting fragments LKPTPEGDL and LKPTPEGDLEIL [36].

Recently D’Souza et al., 2020, suggested that peptides released from BSA have pleiotropic metabolic effects on adipocytes and skeletal myotubes; in particular, they promote adipocyte differentiation and the activation of peroxisome proliferator-activated receptors (PPARγ and PPARδ) responsible for an increase in lipid storage and oxidation. In myotubes, peptides reduced lipotoxicity-induced inflammation, endoplasmic reticulum stress and diacylglycerols accumulation, and enhanced the incorporation of fatty acids into the triacylglycerol pool, thereby countering insulin resistance [59]. Promising sequences for the development of innovative drugs against diabetes were also obtained from WPs of minor species [60,61,62].

Peptides from WPs could be exploited in obesity therapy. After an 8-week treatment period, the oral administration of WP hydrolysate (100 mg/kg body weight) enhanced blood glucose clearance, alleviated hyperinsulinemia and restored the pancreatic islet function for glucose-stimulated insulin secretion in obese mice [63]. In a recent study, peptides exerted a protective role in obesity-induced inflammation [64].

WP hydrolysates can also exert satiating properties [65]. Several of the identified peptides (most of them released from β-LG) act as glucagon-like peptide-1-1, cholecystokinin inducers and DPP-IV inhibitors (Table 1), and have comparable bioactivities to peptides/hydrolysates from other sources (pea, turmeric, beef, olive leaf and grape seed). The advantage for dairy proteins lies in their capacity for substantial consumption as integral components of diverse food matrices. Their incorporation into various formulations may facilitate a reduction in portion sizes and overall food intake over time. However, their efficacy can be lost during gut transit; innovative approaches for protecting orally administered peptides during gastric transit to the small or large intestine are ongoing [65].

Chronic inflammatory responses are associated with the pathogenesis of several NCDs. Peptides from WPs were found to be responsible for the reduction in IL-1β, cyclooxygenase-2 and TNF-α mRNA levels; in addition, novel peptides from buffalo WPs (DQPFFHYN and YSPFSSFPR) exhibited pro-inflammatory mediators iNOS, TNF-α and IL-6 in inflammatory macrophages, indicating a protective mechanism against the cell inflammation damage [53]. Through the regulation of inflammatory cytokine gene expression and the reduction in key inflammatory mediator levels, WP hydrolysate effectively attenuated the inflammatory response in mouse models of colitis [66] and hyperuricemia aggravated by renal inflammation [67]. An extensive amount of the literature also reports the effect of whey peptides on specific (lymphocyte activation and proliferation, antibody production, cytokine expression) and non-specific (functions of macrophages, granulocytes and natural killer cells) immune responses [68,69].

### 2.2. Gut Microbiota Modulation

Several chronic diseases are responsible for changes in the gut microbiota. Alterations in this usually result in worse illness outcomes and undermine therapy efficacy. In light of this, recent studies are focused both on the mechanisms underlying gut microbiota changes and on scouting for strategies that are able to restore it.

LF and its BAPs play a crucial role in promoting and maintaining a functional gut microbiota while preventing gut barrier dysfunction (Table 1). In particular, experimental evidence suggests that these effects are primarily attributed to their strong antimicrobial activity and immunomodulatory functions, which contribute to maintaining gut barrier integrity [70].

Indirect prebiotic effects have also been observed on the growth of probiotics, such as *Bifidobacteria* and *Lactobacilli*; in weaning piglet models, the administration of 0.1 g/kg of a recombinant fusion peptide, constituted by lactoferricin and lactoferrampin, increased the amount of this population in the chyme of the stomach, duodenum, jejunum, ileum, colon and caecum [71].

Several authors demonstrated in mouse models that the BAPs produced from WPs (e.g., PEW) reduced hyperuricemia symptoms [67,72] (Table 1). Xie and colleagues also showed that treatment with α-LA hydrolysates effectively alleviates hypertension-induced intestinal microbiota dysbiosis in spontaneously hypertensive rats [45]. Similarly to LF, peptides from α-LA showed prebiotic effects of gut microbiota [73]. Gut microbiota changes associated with antidiabetic effects were also demonstrated for GMP hydrolysate administration in type 2 diabetes mouse models [74].

Due to their antioxidant properties, WP peptides had an important role in colitis alleviation [66]; furthermore, the administration of WP-derived peptides led to an increased relative abundance of beneficial gut bacteria in mice with enteritis, while significantly suppressing the growth of pathogenic bacteria [66].

### 2.3. Cancer

Emerging research suggests that peptides from WPs can exert anticancer properties by modulating signaling pathways involved in cancer development. BAPs reduced the growth of cancer cells by the dysregulation of the mammalian target of the rapamycin (mTOR) pathway, playing a fundamental role in the regulation of cell growth and metabolic processes. In addition, peptides have been found to modulate the inflammatory response, which plays a decisive role at different stages of tumor development, including initiation, promotion, malignant conversion, invasion and metastasis.

Studies in humans and animals reporting the efficacy of LF in cancer therapy have demonstrated that LF is converted into LfcinB (f17–41) and other peptides during its transit in the gut, and that these latter peptides might display anticancer properties. The mechanisms of action proposed were the following: induction of apoptosis, increase in DNA fragmentation and morphological changes and cell cycle arrest at the G2 phase in breast cancer cells. These effects were attributed to the downregulation of the mTOR signaling pathway and the upregulation of the AMPK signaling pathway [75].

LF-derived peptides (FKCRRWQWRMKKLGAPSITCVRRAF, RRWQWRMKKLG, RRWQWR, RRWQWRMKKLG, FKARRWQWRMKKLGA, GEQELRKCNQWSGLSEGSVT, WSGLSEGSVTCSSASTTEDC and RWQWRWQWR) inhibit the growth, adherence and migration of breast cancer cells, promote apoptosis, induce cell cycle arrest and upregulate the expression of apoptosis-related proteins, thus hindering cancer progression. Likewise, GEQELRKCNQWSGLSEGSVT, WSGLSEGSVTCSSASTTEDC and CSSASTTEDCIALVLKGEAD from human LF exhibit very strong cytotoxicity in several breast cancer cell lines [76] (Table 1).

Researchers have also explored the anti-breast and colon cancer effects of peptide mixtures from WPs of camel and goat [77,78]. Furthermore, investigations have reported that WP-derived peptides are also able to activate specific signaling pathways, such as p38 MAPK and p53 signaling [79].

Recently, Liu et al., 2023, investigated the antitumor properties of a whey-peptide-based enteral diet (MEIN^®^), formulated with cheese whey and multiple nutrients, which was selected to evaluate its anti-tumor effects through animal experiments. The whey-peptide-based enteral diet significantly reduced tumor weight growth, tumor cell proliferation and inflammatory cell infiltration in the spleen and liver of tumor model mice, an effect potentially attributed to its whey peptide components [80].

### 2.4. Other NCDs

Some other NCDs commonly affect people worldwide, including central nervous system diseases. Neuroprotective effects were demonstrated in aged mice model, suggesting a role of WP peptides in the development of novel strategies against Alzheimer diseases [81,82] (Table 1). Yu et al., 2021 [83], found that whey protein peptides can significantly improve the decline in spatial exploration, physical exercise and spatial and non-spatial learning/memory ability related to aging. Their specific mechanism may be related to the reduction in hippocampal neuron degeneration, reduction in neuron apoptosis, improvement in AChE activity, improvement in mitochondrial dysfunction, reduction in the expression of inflammatory factors (TNF-α and IL-1β) in brain tissue, reduction in oxidative stress damage and improvement in BDNF synaptic plasticity protein. Similar results were also reported by Ding et al., 2023, 2024 [81,84]. In addition, the modulation of gut microbes related to Alzheimer’s disease was documented after WPH treatment [82,84].

Wu et al., 2022, reported that the supplementation of whey protein peptides might be an effective and optimal strategy for sarcopenia prevention and treatment. In particular, the results proved that peptides improved muscle loss by promoting muscle protein synthesis in the aged skeletal muscle of galactose(d-gal)-induced aging mice [85].

The effects of whey-derived lactopeptide β-lactolin (GTWY from β-LG) on cognitive performance in mild cognitive impairment were investigated in a clinical trial by Umeda et al. (2024), recruiting a total of 422 middle aged individuals [86]. Previously, the treatment of healthy middle-aged adults (45–64-year-old and 50–75-year-old) with GTWY-rich whey peptides enhanced cognitive performance associated with frontal cortex activity (verbal fluency test, memory, attention; [87,88]). These results showed that long-term intervention with β-lactolin provides a promising strategy for the prevention of diseases of cognitive systems related to aging (Table 1).

Recently, the peptides β-lactotensin (β-LT) and wheylin-1 and -2 with anxiolytic and anti-stress properties were identified from α-LA and β-LG, respectively [89].

Although atopic dermatitis has been associated with milk allergy, hydrolyzed whey-based infant formulas (pHF-W) have been shown to decrease the risk of this disease (AD) in infants; more specifically, the administration of pHF-W both induced oral tolerance and improved skin barrier function, protecting the skin from non-milk-related entries of allergens [90]. A few years earlier, XP-828L, a whey protein extract, also containing BAPs, demonstrated potential benefits also for the treatment of mild to moderate psoriasis [91] (Table 1).

**Table 1:** Impact of whey-protein-derived peptides on NCDSs in in vivo studies.

NCDS	Disease	Native Proteins	Peptides/Hydrolyzed	In Vivo Model/Clinical Trials	Biological Activity	References
Metabolic disorders	Obesity	β-LG	LIVTQTMKG, (f1–9) (oral consumption 1 mg/kg of body weight (BW)	Mice model ddY mice	Decreases food intake and plasma ghrelin (satiety activity)	[65]
			HIRL (β-lactotensin)	C57BL/6J mice	Delay in gut transit, reduction in food intake (I.p. injection, oral consumption) (satiety activity)	[65]
			VAGTWY	C57BL/6 mice	Lowers plasma glucose level in oral glucose tolerance test (satiety activity)	[65]
		α-LA	α-LA hydrolysate	High-fat diet (HFD)-induced obese mice	Reduction in the levels of inflammatory cytokines (IL-6, TNF-α and LPS); reduction in obesity-associated systematic inflammation and endotoxemia. Increase in Bacteroides/Firmicutes ratio; reduction in pathogenic bacteria load; increase in short-chain fatty acid (SCFA)-producing bacteria	[65]
			Administration of peptide DQW	(HFD)-induced NAFLD mice	Increase in Bacteroides/Firmicutes ratio; reduction in pathogenic bacteria; increase in SCFA-producing bacteria; improvement in intestinal barrier integrity and inflammation	[70]
		BSA	AFKAWAVAR (Albutensin A)	dYY mice	Delay in gut transit, reduction in food intake (I.p. injection) (satiety activity)	[65]
		Whey proteins (WPs)	2% and 4% WPH for 12 weeks	HFD-fed obese mice model with significantly imbalanced redox status and reduced bone mass	Improvement in HFD-induced bone loss, mainly through their antioxidant and osteogenic capacity by activating Runx2 and GSK-3β/Nrf2 signaling pathway	[58]
	Hyperuricemia	α-LA	Oral supplementation of α-LA hydrolysates	Potassium oxonate- and hypoxanthine-induced hyperuricemic mice	Increases the abundance of some SCFA-producing bacteria; decrease in the growth of hyperuricemia- and inflammation-associated genera	[70]
		β-LG and uncharacterized protein	ALPM (30 mg/kg BW) and LWM (30 mg/kg BW)	Sprague–Dawley rats	Reduction in serum uric acid levels and xanthine oxidase activity	[92]
	Dysbiosis	α-LA	Oral gavage of α-LA hydrolysates under 3 kDa (100 mg/kg BW) and VGINYW (5 mg/kg BW)	Spontaneously hypertensive rats (SHRs)	Reduction in hypertension-associated intestinal microbiota dysbiosis; preservation of gut microbiota biodiversity and modulation of SCFA-producing bacteria	[45]
	Colitis	WPs	500 mg WPH/kg day for 33 days	Dextran sulfate sodium (DSS)-induced colitis in mice	Regulation of mRNA expression of inflammatory cytokines; strengthening of tight junctions; modulation of oxidative stress levels; gut microbiota regulation	[66]
	Diabetes	β-LG	LIVTQTMKG	Alloxan-induced type 1 diabetic mice	Reduction in blood glucose levels and glycated serum proteins; increase in glucose transporter-2 expression; protection of injured β cells by suppressing apoptosis; rescuing of Ki67 immunoreactivity through IRS2/PI3K/Akt signaling; increase in the phosphorylation of FOXO1; and upregulation of PDX-1 expression, in turn, resulting in increased insulin secretion	[93]
		Glycomacropeptide (GMP)	8-week GHP hydrolysate dietary supplementation	HFD-fed and streptozotocin-induced type 2 diabetic C57BL/6 J mice	Hypoglycemic activity; reduction in dyslipidemia and inflammation; increase in the Bacteroidetes/Firmicutes ratio	[74]
Cardiovascular diseases	Hypertension	WPs	400 mg WPH/kg BW; 240 mg WPH/kg BW	SHR	Reduction in renin concentration and systolic blood pressure	[94,95]
		WPs	WPH 500 or 1000 mg/kg BW	SHR	Reduction in renin concentration and systolic blood pressure; increase in *Akkermansia*, *Bacteroides* and Lactobacillus abundance and, consequently, increase in the amount of promoted high-SCFA content in feces	[44]
	Hypertension	LF	LRPVAA (1 nmol/kg BW)	SHR	Reduction in systolic blood pressure	[40]
		LF	RRWQWR (f20–25) and WQ (120 mg/Kg of BW)	SHR	Reduction in systolic blood pressure	[40]
		BSA	KFWGK (5 g kg^−1^; oral administration	SHR	Long-lasting antihypertensive effect via cholecystokinin (CCK)-dependent vasorelaxation	[96]
			Papain hydrolysate of BSA	SHR	Reduction in systolic blood pressure over a 24 h period	[97]
		WPs	WPH containing IW and WL (5 and 50 g)	Healthy volunteers	Reduction in plasma ACE activity	[47]
Neurological diseases	Neurodegeneration	LF	SVDGKEDLIW oral administration for 6 weeks (0.1 g/kg BW)	D-galactose treated Kumming mice	Increase in antioxidant enzyme (SOD and GSH-PX) activity	[55]
	Neurodegeneration	WPs	0.3–3.0 g WPH/kg BW for 30 days	D-galactose-treated mice	Reduction in the decline in aging-related spatial exploration, body movement, and spatial and non-spatial learning/memory ability. Reduction in the degeneration of hippocampal nerve cells; apoptosis of nerve cells; increase in the activity of AChE; reduction in the expression of inflammatory factors (TNF-α and IL-1β) in brain tissue and oxidative stress injury; increase in the expression of p-CaMKII and BDNF synaptic plasticity protein	[83]
		WPs	30–100 mg WPH/kg BW	C57BL/6 J for 30 weeks	Modulation of the morphology and organization of hippocampal cells; reduction in inflammation and oxidative stress; modulation of gut microbiota	[82]
	Age-related cognitive declines	β-LG	1 g of whey peptide, which included 1.6 mg of β-lactolin of GTWY fo2 12 weeks	Healthy adults (45–64 years old and 50–75 years old)	Improvement in cognitive performance associated with frontal cortex activity (verbal fluency test, memory, attention)	[87,88]
			β-lactolin (1.8 mg daily) or placebo for 24 weeks	Adults (≥50 years old)	Improvement in Montreal Cognitive Assessment (MoCA-J) scores for cognitive function	[86]
	Alzheimer’s disease	WPs	30 and 100 mg WPH/kg BW for 140 days	APP/PS1 mice	Improvement in memory impairments through bidirectional effects of the gut microbe–SCFA–brain axis	[84]
			10 and 100 mg WPH/kg for 10 days	Scopolamine-induced cognitive impairment mice model	Reduction in neuronal damage	[81]
	Anxiety	β-LG	β-lactotensin (HIRL, f146–149) 1–10 mg/kg BW oral administration	C57BL/6 and ddY mice	Interaction with neurotensin (particularly NTS2) and dopamine receptors	[89]
Cancer	Breast cancer	WPs	WPH	Female mice	Protection against DNA damage in rat mammary glands compared to casein protein	[75]
	Colon cancer	WPs	WPH	Female mice	Inhibition of colon cell cluster development	[75]
	Fibrosarcoma	LF	LFcinB	Individuals	Reduction in the size of solid Meth A fibrosarcoma	[98]
Skin disease	Wounds	Human LF and bovine β-LG	IAENRADAV and GSPSGQKDLLF from human LF; LDTDYKKY from β-LG	Infected wound model	Anti-inflammatory activity, increase in collagen synthesis and deposition, angiogenesis and tissue regeneration	[99]
	Psoriasis	WPs	Whey protein extract XP-828L (2.5 g, twice a day, over 112 days)	Human subjects with mild to moderate psoriasis	Reduces symptoms of psoriasis	[91]
	Atopic dermatitis (AD)	WPs	Partially and extensively WPH formula	Children (6 months of age)	Reduction in the incidence of AD and allergic manifestation	[100]

## 3. Other Biological Properties of BAPs

### 3.1. Antimicrobial, Antibiofilm Activities

Antimicrobial resistance (AR) is currently one of the major global public health threats. The 2022 Global Antimicrobial Resistance and Use Surveillance System (GLASS) report highlights alarming resistance rates among prevalent bacterial pathogens [101]. Antimicrobial peptides (AMPs) show several advantages that make them a promise in the development of new drugs. They comprise short sequences with broad-spectrum action, low resistance, water solubility, synergistic effects with common antibiotics, multi-target activities and an absence of toxic effects on gut microbiota [102,103]. The mechanisms of action of AMPs include cell membrane damage, inhibition of macromolecule synthesis, damage to organelles resulting in DNA fragmentation and inhibition of enzyme activity [102]. As an example, peptides from LF bind to the cell surface, release LPS, disrupt the outer membrane and form ion channels on membranes [104]. Infections by MDR pathogens and underestimated bacteria aggravate the severity of NCDs and counteract NCD treatment [105,106].

Bovine α-LA, β-LG and LF are important sources of antimicrobial peptides with high activity against several genera and microorganism species; the identified activities and related spectrum of action are widely reported in available on-line databases [103]. Thus, recent studies are focused on the improvement in WP hydrolysis by selected strains or enzymes in order to increase the yield of antimicrobial peptides, as well as their activity [49,107,108,109,110,111,112].

In addition, due to the high significant inter-species diversity of WPs [113], the research of novel antimicrobial sequences from non-bovine WPs is currently being pursued [114,115,116]. Recently, Liu et al. (2024) [117] identified the fragments TPEVDDEALEK and LIVTQTMK (f141–151; f19–26) from β-LG isolated from buffalo colostrum with an antimicrobial activity against *E. coli* and *S. typhimurium*, *S. aureus* and *L. monocytogenes.* The authors also demonstrated the resilience of LIVTQTMK to variations in pH, temperature, osmotic pressure and enzymatic digestion. Bioinformatic analysis of presumptive antibacterial peptides from camel milk protein hydrolysate allowed one to identify the albumin-derived fragment WSVGH, binding to specific protein targets involved in the growth and development of bacteria (penicillin-binding protein 1a, aminoacyl-tRNA synthetase, dihydrofolate reductase) [118]. Sheep’s milk fermented with *L. fermentum* KGL4 exhibited promising antioxidant and antimicrobial activities due to the release of bioactive sequences, including the LDQWLCEK (f134–141) and KADEKKFW f(151–158) from α-LA and albumin, respectively [115].

Among the healthy properties (inhibitory activity against pancreatic lipase, α-amylase, ACE) for goat WP hydrolysate, a high antimicrobial activity against *E. coli*, *S. aureus* and *B. cereus* was reported; additionally, the peptides demonstrated significant cytokine inhibition and low toxicity toward the HT-29 cell line [119] by demonstrating its potential for developing value-added products with excellent safety standards [22,120,121,122].

Wang et al., 2021 [123], also investigated the antimicrobial activities of deer LF-encrypted peptides lactoferricin (LFcinB) and lactoferrampin (Lfampin). Deer LFcinB and Lfampin shared 72% and 90% similarities with bovine LFcinB and Lfampin, respectively. This difference in sequence homology caused changes in antimicrobial activity compared to bovine-derived peptides. In particular, deer LFcinB was found to be more effective against *L. acidophilus* ATCC 4356 than bovine LFcinB; by contrast, bovine LFcinB and LFampin were more effective against *E. coli* ATCC 25922 than deer LFcinB and Lfampin [123].

Pathogens increase their resistance to antimicrobials and persistence in the environment when they are in the form of biofilms [124]. A biofilm is a microbial community embedded in a self-produced matrix of extracellular polymeric substances (EPSs) adhering to a biotic or abiotic surface. AMPs derived from WPs offer a novel therapeutic option to combat biofilm; the antibiofilm activity of LF-derived peptides is the most studied and is still yielding novel insights [125,126,127,128,129,130,131]. The mechanism of action of these peptides was investigated by Quintieri et al., 2019 and 2020, by a comparative proteomic analysis of treated and untreated *Pseudomonas* spp. cells [132,133]; the downregulation of proteins involved in exopolysaccharide synthesis, as well as the induction of biofilm dispersal factors, was found in treated cells. In addition, peptides reduced swimming- and twitching-motility-related proteins [132].

Camel milk whey hydrolysate also inhibited biofilm formation by *P. aeruginosa* PAO1 and methicillin-resistant *S. aureus* [55].

Biofilms are regulated by a complex cell–cell communication system, well known as *quorum sensing.* This mechanism of intra- and inter-cell communication, mediated by signal molecules (autoinducer-2, N-acyl-homoserine lactone, auto-inducing peptides, diffusible signal factors), synchronizes gene expression in response to population cell density, hence coordinating community behaviors to counteract unfavorable stress conditions [134]. Although the exploitation of WP-derived peptides as quorum quenchers is suggested in the current literature [135,136], to the best of our knowledge, only the synergistic antimicrobial action of LF-derived peptides and quorum quenching enzymes is reported [137].

To sum up, WP-derived peptides showed high potential as antimicrobial and antibiofilm agents, although their exploitation in the food and clinical sectors is still limited and mainly tested in laboratory-scale models/studies. Recently, multi-target sequences derived from human LF (IAENRADAV and GSPSGQKDLLF) and human β-LG (LDTDYKKY) also accelerated the recovery of an infected wound model thanks to the anti-inflammatory and regeneration properties of AMPs by highlighting their potential for developing wound-healing therapies [99].

Vergis et al., 2020, evaluated the in vitro antimicrobial and antibiofilm efficacy of lactoferricin (f17–30) against biofilm-forming multi-drug-resistant (MDR) *E. coli* and, subsequently, the in vivo antimicrobial efficacy was assessed in a *Galleria mellonella* larval model. The results of the study showed that peptides produced an increased survival rate of larvae, a lower bacterial count and reduced histopathological changes, while producing enhanced immunomodulatory effects [138]. The effects of WP hydrolysates on *L. monocytogenes* virulence gene expression were recently explored in simulated gastrointestinal conditions [139].

WP-derived AMPs have been applied in the food sector as an alternative to preservatives in order to inhibit foodborne microorganisms as well as spoilage microflora (Table 2). Quintieri et al. reported that a bovine LF hydrolysate produced by digestion with pepsin inhibited the growth of bacteria contaminating mozzarella cheese during cold storage [140] and their spoilage traits [141]. The efficacy of bovine LF hydrolysate was also checked in *Pseudomonas* spp.-contaminated ready-to-eat vegetables [142]. A high-throughput analysis of bacterial growth also highlighted an underestimated pathogenic behavior of these species, whose occurrence was previously reported in patients affected by chronic diseases [133].

The application of WP hydrolysate with antimicrobial activity in a soft-cheese-based model inhibited *L. monocytogenes* growth but stimulated its virulence determinants [143].

The addition of whey hydrolysates with antioxidant and antimicrobial activities into a beverage represented a concrete link between food science and therapeutic nutrition [144].

**Table 2:** Application of BAPs from WPs in food sector.

Native Protein	Treatment of Food Models		Biological Activity	References
Lactoferrin (LF)	Dairy products	Preservation of milk supplemented with peptic hydrolysate (≤2 mg/mL) under limiting conditions (4 °C and pH 4.0)	Antimicrobial activity against *Escherichia coli* O157:H7 and *Listeria monocytogenes*	[145]
		Preservation of high-moisture mozzarella cheese stored in governing liquid supplemented with pepsin-digested hydrolysate (LFH; 10 mg/mL) under cold storage period.	Antimicrobial activity against naturally contaminating microflora; antimicrobial activity against spoilage bacteria belonging to *Pseudomonas* spp.; inhibition of pigment release by *P. lactis*	[140,141,146,147,148]
	Fruits and vegetables	Replacement of fungicides by the application of LFcinB and LF f(17–31) on mandarin fruits	Antimicrobial activity against *Penicillium digitatum*	[149]
		Application of hydrolysate (50 mg/mL) and LfcinB f(17–31) (3 mg/mL) in ready-to-eat vegetables	Antimicrobial activity against spoilage *Pseudomonas* spp.	[140,142,146,147,148]
	Wine	Application of pepsin hydrolysate (1–10 mg/mL) and LfcinB f(17–31) during winemaking processes without compromising wine attributes	Antimicrobial activity against *Saccharomyces cerevisiae* and other spoilage wine yeasts (*Cryptococcus albidus, Dekkera bruxellensis, Pichia mem branifaciens, Zygosaccharomyces bailii* and *Zygosaccha romyces bisporus*) and bacteria (*Levilactobacillus brevis, Lactobacillus hilgardii, Pediococcus damnosus* and *Oenococcus oeni*)	[150,151]
	Meat	Application of pepsin-digested lactoferrin in contaminated chicken fillet (0.5 mg/g) under hydrostatic pressure (0, 300, 400 and 500 MPa) for 10 min at 10 °C	Antimicrobial activity against *E. coli* O157: H7 and *P. fluorescens*	[152]
Whey protein isolate (WPI)	Meat	Application of hydrolyzed whey protein isolate (2%) in pork patties before cooking and subsequent cold storage for 1 week	Antioxidant activity of peptides inhibited lipid oxidation	[153]

### 3.2. Antiviral Activities

WPs and related BAPs were reported to exert antiviral actions against different enveloped and non-enveloped viruses. The most important antiviral properties have been documented for native LF and related peptides (LFcinB and LFampin); in most cases, the latter showed an augmented activity compared to the native protein [154,155]. β-LG, α-LA fragments and their chemical modifications displayed antiviral properties against human herpes simplex virus type 1 (HSV-1) [156]) An in silico approach also suggested the β-LG-derived peptides ALMPHIR and IPAVFK as potential candidates to fight SARS-CoV-2 [157]. Later, Gambacorta et al. (2022) [14] validated in vitro the inhibitory activity of the shorter sequences IAEK, IPAVF and MHI against the SARS-CoV-2 3C-like protease; these peptides also exhibited ACE-inhibitory activity [17]. No inhibitory activity was instead reported for human rhinovirus 3C protease [14]. Most of the known mechanisms of action involve interactions of the antiviral agent with host cell receptors or with the viral genomes preventing viral entry and replication in cells [154].

## 4. Safety Considerations

BAPs from WPs derived by natural enzyme hydrolysis or fermentation from food sources are usually considered as non-toxic and harmless [16,94,120,158]. As peptides are intrinsic in nature, their target/receptor-binding affinities are specific, leading to higher selectivity and efficacy and few toxic reactions. In the human body, the cleavage of peptides after enzymatic digestion releases amino acids, resulting in few incidents of toxicity and drug–drug interactions. In addition, the short half-life of peptides and the fact that their metabolites do not accumulate into the tissues reduce the risk of metabolic toxicity. Peptides are also potentially less immunogenic due to their small size [9].

Toxicology studies of bovine LF, LF hydrolysate and the well-known peptide LFcinB showed that no adverse effects were displayed [120]; by contrast, cytoprotection against oxidative stress was found [22].

Favorable indications of the nutritional safety of a specific protein hydrolysate from WP concentrate, deemed safe for its use in infant formula, have very recently been reported [159]. Notably, the EFSA panel members concluded that the protein hydrolysate was nutritionally safe and a suitable protein source for use in infant formulae as long as the formula in which it is used contains a minimum of 2.0 g/100 kcal protein and complies with the compositional criteria of Regulation (EU) 2016/127 and the regulated amino acid pattern.

Although no experimental data have been provided on the nutritional safety and suitability, a hydrolysate from WP concentrate was approved in 2022 for consumption by infants based on fact that the protein source is considered to be nutritionally safe [159].

Overall, whey-peptide-based products addressing consumption have to meet the requirements of the EC 1924/2006 regulation with health claims [160] (Available online: https://eur-lex.europa.eu/legal-content/EN/TXT/PDF/?uri=CELEX:32006R1924, accessed on 19 February 2025).

Considering this latter consideration, further investigations are needed to provide more insights into the toxicological aspects and allergic reactions induced by BAPs from WPs. Amino acid racemization, peptide modification/derivatization and Maillard reaction likely to occur during peptide preparation can lead to the formation of numerous potential hazardous compounds [161]. Therefore, additional studies on the administration dosages, frequency and length of use carried out by experimental models and randomized clinical studies should be performed in the future to actually assess the safety of BAP products.

Since allergenic reactions are most common by ingesting WPs, special attention will be given to this aspect in the following paragraphs.

### Whey Protein Allergenicity

Milk proteins are an ideal choice for formulating various products due to their high digestibility, abundant content of essential amino acids and excellent functional properties [162]. A large number of studies have confirmed that casein, β-LG and α-LA are the main allergens found in milk; however, caseins are considered to be poorly immunogenic compared to WPs due to the absence of a tertiary structure that exposes them to a higher degree of hydrolysis during digestion [163,164,165,166].

As is well known, milk allergies can be avoided by excluding the allergenic food from the diet. Nevertheless, in recent years, diverse technological strategies have been investigated and devised to reduce the allergenicity of milk proteins [167,168,169,170,171].

Notably, in the last two decades, studies have been directed toward developing methods to produce hypoallergenic milk concentrates that might allow for the consumption of milk components by milk allergy sufferers [172,173].

Due to the high value of milk components, to avoid newborns and toddlers being deprived of important elements carried through the milk in cow’s milk allergic individuals, a hypoallergenic milk substitute would be beneficial to introduce during infancy and childhood. Hypoallergenic formulas represent nutritionally valuable alternatives to the consumption of milk. These formulae are produced through the enzymatic hydrolysis of different sources, such as bovine casein or whey fractions, followed by further processing, including heat treatment and/or ultrafiltration as described by Quintieri et al., 2017 [171]. Depending on the extent of protein hydrolysis, the resulting products can be classified as ’extensively’ or ’partially’ hydrolyzed. Upon their production, the reduction in allergenicity should be assessed in vitro and in vivo. While casein proteins are more prone to being degraded into small peptides during human digestion, thanks to the multiple target sites of digestive enzymes, some interesting investigations have been carried out on the whey fraction (considered highly allergenic and gastro-resistant proteins) [174]. Conventional food processing technologies able to decrease the allergenicity of milk proteins include heat treatment, fermentation and enzymatic hydrolysis [175,176].

New technologies available include high-pressure processing, irradiation and ultraviolet and infrared radiation [177,178,179]. Among the different approaches described in the literature, enzymatic hydrolysis proves to be the most effective in removing the immunoreactivity of milk proteins. Wroblewska and Troszynska (2005) were among the first authors reporting that the immunoreactivity of the hydrolysate obtained by the alkaline protease hydrolysis of WPs was significantly reduced [180].

So far, a multitude of enzymatic-based workflows have been devised for WP allergenicity reduction in order to push toward the re-introduction of these nutritionally valuable proteins in the diet of allergic consumers. In this regard, optimization of a two-step hydrolysis method was described and reported to effectively reduce WP allergenicity [181].

According to the available data, about 97% of hydrolyzed peptides from α-LA and β-LG did not contain allergenic epitopes upon undergoing treatment with trypsin and flavourzyme. In vitro and in vivo allergenicity assessments confirmed that this method was effective in reducing the allergenicity of WPs; it was also demonstrated that, compared to the common milk powder, the resulting hypoallergenic formula induced lower levels of basophil degranulation [181]. It was reported that submitting MPC to extensive hydrolyzation with EMPHs for 4 h with four EMPHs ((AX, Alcalase-Protamex), (AD, Alcalase-Protease A 2SD), (AE, Alcalase-Flavourzyme), (AH, Alcalase-ProteAXH)) caused different degrees of hydrolysis in the range comprised between 12 and 19% and assessed by peptidomic analysis [182]. This highlights that the efficiency of the hydrolysis process strictly depends on the specific type of enzyme and the incubation time fixed.

## 5. Role of Whey-Derived Peptides in Emerging Therapeutics for Immune Tolerance: The Tolerogenic Effect

**Figure 2:**
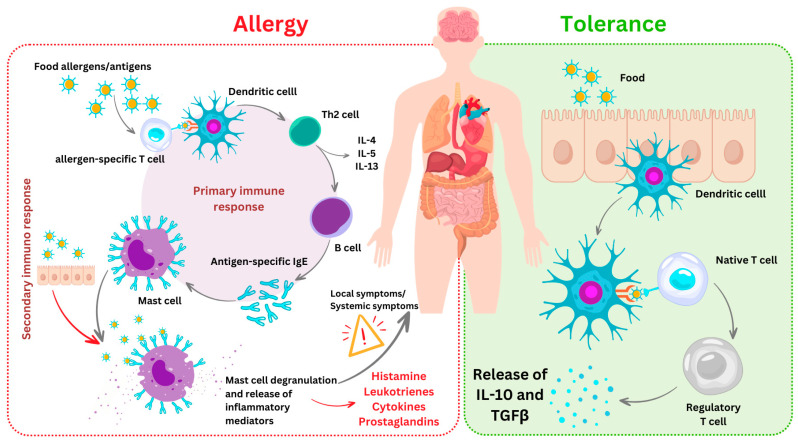
Comparative representation of the tolerogenic *versus* allergic response to food antigens.

Food allergy represents an increasingly prevalent immune disease with potential life-threatening implications, and is the result of a defect in the existing tolerance [183,184]. Oral tolerance (OT) to food proteins is a natural immune process in which the immune system recognizes common antigens as harmless [185]. When this mechanism does not work correctly, the harmless compound is recognized as a menace for the body and is attacked by the immune system, triggering an adverse immunological reaction as displayed in Figure 2.

The gastrointestinal tract contains lymphocytes, as well as unique antigen-presenting cells with specialized functions. If the antigen is initially encountered during its transit along the gut, this system generates a robust T-cell-mediated hyper-responsiveness called oral tolerance. A single epithelial layer separates this antigenic load from the lymphocytes, antigen-presenting cells (APCs), stromal cells and other immune cells comprising the mucosal-associated lymphoid tissue (MALT). Within the MALT, distinct populations of dendritic cells (DCs) engage with dietary antigens and influence the outcome of the subsequent adaptive response, determining whether immunity or tolerance is induced. When the initial antigen exposure occurs via the GI tract, a robust T-cell-mediated suppression, known as oral tolerance, is established [186] (Figure 2).

Food allergy is among the clinical disorders that occurs from a failure of this system [187]. Regarding their tolerogenic effect, it has been suggested that selected milk protein hydrolysates may be able not only to avoid displaying allergic symptoms in cow-milk-allergic (CMA) children due to the destruction of IgE epitopes but might also have immune-modulating properties, like the induction of T cell tolerance and the prevention of sensitization [186,188]. An increasing number of data suggest a potentially different impact on immune tolerance acquisition induced by different formulas available for CMA management [189,190,191,192,193].

In a recent paper authored by Paparo et al. [194], a comparative study was carried out to evaluate the tolerogenic effect elicited by protein fractions of different formulas available for the dietary treatment of CMA, namely the extensively hydrolyzed whey or casein formula (EHWF, EHCF), hydrolyzed rice formula (HRF), soy formula (SF) and amino-acid-based formula (AAF). These formulas were also subjected to an in vitro infant gut-simulated digestion, and the findings showed that the EHCF was able to elicit a tolerogenic effect through a partial epigenetic modulation of the FoxP3 gene. As explained, the gut-barrier-related non-immune mechanisms (such as epithelial permeability and mucus thickness) are considered relevant in preserving immune tolerance [195]; therefore, the loss of gut barrier integrity can produce an increased antigen uptake, promoting a Th2-type allergic response by the activation of type 2 innate lymphoid cells (ILC2s), mast cells, basophils and dendritic cells (DCs) [196]. The authors demonstrated that EHCF-derived protein fractions could improve gut barrier integrity, increasing occluding and ZO-1 and Muc5AC expression in human enterocytes, therefore promoting a potential tolerogenic effect. The other formulas (EHWF, HRF and AAF) were unable to modulate these components of the gut barrier, instead reporting that they promoted an increase in TSLP and/or IL-33 production.

Other studies have shown that formulas made from the EHWF and EHCF, containing only peptides with a molecular weight of less than 5000 Da, are suitable for cow’s-milk-allergic patients, as these proteins have lost their antigenic and allergenic potential [197,198]. In contrast, partially hydrolyzed formulas were shown to keep part of their antigenicity, including the capacity of IgE antibody induction [197,199], and to keep a tolerogenic potential, whereas extensively hydrolyzed proteins did not retain such potential [200,201]. Partially hydrolyzed formulas are recommended for infants at high risk of atopy when breastfeeding is discontinued. These formulas contain protein fragments of various sizes, but may still include residual non-hydrolyzed proteins [201]. Therefore, it is challenging to evaluate the relative contribution of these different products in the tolerance process and/or to identify the specific fragments that are essential.

A paper authored by Adel-Patient et al. [174] reports a study demonstrating the immunomodulatory potential of fragments derived from the cow’s milk allergen bovine β-LG assessed in a mouse model, to be promoted for oral tolerance [174]. In this paper, the immunomodulatory potential of a further allergic sensitization of β-LG, either as a native protein or after chemical denaturation, and of the corresponding CN hydrolysates, was analyzed. Overall, these results show that, in the BALB/c mouse model, partially hydrolyzed β-LG derived from intact β-LG can prevent further sensitization, with greater efficiency when disulfide bridges are maintained between the peptides formed by the hydrolysis. This indicates that the immunomodulatory potential is predominantly influenced by the combination/association of the peptides generated, including the larger ones produced through partial hydrolysis. Such complex compounds may be found in commercially available partially hydrolyzed whey formulas currently used as dietary products, along with potential low levels of intact native or denatured proteins. Both factors may contribute to the observed tolerogenic potential, with relevant synthetic peptides also being evaluated.

Recently, Tian et al. (2023) and Zhang et al. (2023) have described a strategy for the prediction, identification and application of β-LG hydrolysates with oral immune tolerance, which was established using mass spectrometry combined with bioinformatics, also involving T cell proliferation tests and animal experiments. The trypsin hydrolysate of β-LG with abundant T cell epitopes exhibited significant oral tolerance, paving the way for developing an approach for allergy prevention in the future. It was highlighted that β-LG hydrolysates prepared by papain and protamex proteases contained fewer T cell epitope peptides but had good oral tolerance activities. Although the neutral protease hydrolysate contained T cell epitopes, the results proved that they did not induce immune tolerance in mice [202,203].

## 6. Current Drawbacks and Challenge in Whey Peptide Applications

Exploiting BPAs from WPs instead of other bioactive compounds in drug discovery offers several advantages. Some peptide sequences (e.g., those derived from LF) are analogues to those involved in innate immunity, both systemically and locally [204]; BPAs can exhibit multi-target activities [17]; in order to improve their pharmacological properties, they can be more easily modified, using both chemical and biological methods. However, like other natural bioactive compounds, their digestion, absorption and metabolism in the human body are still a bottleneck limiting their wide application. Although, in the last few decades, the number of peptide-based drugs has steadily increased [205], to the best of our knowledge, no whey-derived peptides were approved [9]. Unlike WPs, clinical trials for the pharmaceutical use of whey peptides in the treatment of NCDs are indeed limited (Table 1) and need to be increased to allow for their exploitation in drug development.

The routes of administration affect the stability and bioavailability of whey peptides in human blood, reducing biological effects and thus hindering their exploitation for drug development. As highlighted in Table 1, whey-derived BAPs are usually administrated by oral ingestion. Due to the enzymatic hydrolysis in the gastrointestinal tract, oral delivery represents an obstacle to the reproducibility of healthy properties from in vitro to in vivo studies; whey peptide bioavailability is also limited due to permeation by the mucus-covered gut barrier. To escape proteolytic activity by digestive enzymes and induce the expected effects, large doses of BAPs or innovative formulations are usually administered [206]. Peptide stabilization by the chemical modification of the molecular structure of active sequences by polymer conjugation, cyclization to rigid structures [207,208] and encapsulation in nanoparticles represent further strategies for preventing enzymatic hydrolysis in the gastrointestinal tract [209].

It should be noted that an altered intestinal environment, for example, provoked by ulcerative colitis or colon cancer, as well as pharmacological treatments or endogenous hormones, could influence peptide absorption and reduce its biological effects. As a result, physiological factors should be taken into account in both in vivo studies and in future clinical therapies [210].

Dairy protein hydrolysates also exhibit a bitter taste, high hygroscopicity, low solubility and physiochemical instability; these characteristics reduce consumer acceptance and then limit their application in the food industry. The research of methods able to minimize negative attributes of whey peptides, allowing for wide usage, is another challenge to be overcome [209].

Regarding the BPA intake through food, the interaction of peptides with the food matrix should be considered and more studies on the bioaccessibility and bioavailability of active peptide sequences should be performed. The formation of carbohydrate–peptide adducts (Maillard reaction), metal–peptide complexation, lipid–peptide interactions, phenolic–peptide interactions, changes in health-promoting bioactivities and the formation of toxic species [211] were reported by Lacroix et al. [212] to cause a reduction in the antioxidant activity of whey peptides after incorporation in a matrix containing inorganic salts and glucose. Thus, factors affecting their production and the appropriate food processing should be carefully considered to preserve the biological function of BPAs. Also, the manufacturing process over time can affect reproducibility in the molecular composition and functional properties of BAPs. Bourdeau et al., 2021 [169], demonstrated that the peptide size distribution from 27 batches of a whey-based infant formula produced over 10 years was conserved over the time; likewise, no changes in sensitization and reproducibility in oral tolerance were observed in animals by analyzing trials from different years. In order to satisfy the right balance in size, sequence, structure and dose, tolerogenic peptides have to meet the European Commission Directive 2006/141/EC [160] (available online: https://eur-lex.europa.eu/legal-content/EN/TXT/PDF/?uri=CELEX:32006L0141, accessed on 19 February 2025).

Despite the wide existing literature, the bioaccessibility and bioavailability of BPAs from WPs in the human body is still an unexplored field; thus, future research should address filling this gap to allow for a wide usage of whey peptides in both the pharmaceutical and food industry.

Over the past 20 years, several clinical trials have tested different WP intake levels based on patients’ clinical conditions and the type of disease [213]. In cancer treatment, 30 g for 6 months led to tumor regression in two out of five patients and suggested increased glutathione levels in healthy cells and decreased levels in cancer cells. In stage 4 cancer patients, 40 g for 6 months resulted in 16 out of 20 survivors, with enhanced natural killer cell function, increased glutathione, improved hemoglobin and hematocrit levels and a better quality of life [214]. A recent study by Bumrungpert et al. [215] on 42 patients with various non-metastatic cancers (breast, lung, colon, rectum, stomach, biliary, pancreatic cancer and lymphoma) examined the effects of 40 g of whey protein isolate (WPI) with zinc (2.64 mg/day) and selenium (0.76 mg/day). After 6 and 12 weeks, improvements were observed in protein status, oxidative stress, inflammation, immune function, nutritional status and quality of life. Similar results were also reported by Storck et al. [216], who studied the effect of a leucine-rich supplement combined with nutrition and physical exercise in 52 advanced cancer patients. Patients received Sponsor Sport Food^®^ (50 g BID for 3 days, then once daily for 4 days), along with physical exercise (three times per week) and nutrition counseling. After three and six months, improvements were observed in physical function, nutritional status and dietary intake. However, to the best of our knowledge, few investigations have been carried out to establish whey peptide intake levels in subjects affects by NCDs (Table 1); this lack of investigations opens further questions in this field.

## 7. Conclusions and Perspectives

In drug discovery, peptides exhibit unique properties that resemble some of small-sized drugs as well as attributes of large proteins and other biologics. In the human body, most metabolic and signaling factors (such as enzymes, hormones and immune response mediators) are peptide-based. Peptides have indeed demonstrated to be an important class of drugs capable of simulating various natural pathways and hormonal therapies thanks to the efficacy of tissue penetration, also facilitated by their small size.

Besides their nutritional and technological properties, WP-released peptide sequences endowed with healthy and multi-target activities have been reported with various biological effects (antioxidant, anti-inflammatory, immunomodulatory, etc.) and related mechanisms of action. Based on their potential as therapeutics, the use of whey-derived peptides for the prevention and treatment of lifestyle-related diseases is encouraged, and peptide-based drugs are currently being explored. Lifestyle-related diseases include several chronic diseases (also known as communicable diseases, NCDs) and represent a major concern in the twenty-first century, with millions dying worldwide each year due to chosen lifestyles and associated complications (obesity, diabetes, hypertension, etc.).

To highlight their potential as peptide-based drugs, this review has provided a deep discussion on the current state of the art related to the effects of BAPs from WPs in the prevention and treatment of NCDs. Novel peptide sequences and target activities (such as antisatiety and neuroprotective activity) with special regard to in vivo applications have also been reported.

Finally, a careful look at BAP safety was given since allergic reactions and toxicity cannot be completely excluded. Current scientific opinions consider BAPs safe and suitable for human consumption due to the safety of native proteins and the fact that experimental data are often lacking.

Complementing these considerations is the increasing evidence of the tolerogenic effects of BAPs from WPs in emerging therapies for immune tolerance.

On the other hand, effective strategies used to prevent allergic reactions to food have become a public health priority. Beyond breastfeeding, which remains a key pillar of primary allergy prevention, other nutritional interventions, including the use of whey-based and partially hydrolyzed formulas in non-breastfed infants, have proved to play an important role in food allergy prevention.

Recent studies emphasize the importance of desensitization and tolerance induction through oral and epicutaneous immunotherapy. However, further research is needed to determine the most effective strategies for food allergy prevention at the population level. Moreover, the broader implementation of food allergen immunotherapy may lead to improved health outcomes and enhance the quality of life for families affected by food allergies. It is worthy noting that, although desensitization therapies for food allergies have shown to be effective, many practical barriers restrict their general use.
